# Proliferative Role of Kv11 Channels in Murine Arteries

**DOI:** 10.3389/fphys.2017.00500

**Published:** 2017-07-12

**Authors:** Vincenzo Barrese, Pilar Cidad, Shuk Y. Yeung, Jose R. López-López, Alister J. McNeish, Susumu Ohya, Maria T. Pérez-García, Iain A. Greenwood

**Affiliations:** ^1^Vascular Research Centre, Institute of Molecular & Clinical Sciences, St George's University of London London, United Kingdom; ^2^Departmento de Fisiología, Universidad de Valladolid Valladolid, Spain; ^3^Reading School of Pharmacy, University of Reading Reading, United Kingdom; ^4^Department of Pharmacology, Kyoto Pharmaceutical University Kyoto, Japan

**Keywords:** K^+^ channels, *ether-a-go-go related* gene, Kv11, arterial smooth muscle, proliferation

## Abstract

K^+^ channels encoded by the ether-a-go-go related gene (ERG1 or KCNH2) are important determinants of the cardiac action potential. Expression of both cardiac isoforms (ERG1a and ERG1b) were identified in murine portal vein and distinctive voltage-gated K^+^ currents were recorded from single myocytes. The aim of the present study was to ascertain the expression and functional impact of ERG channels in murine arteries.

**Methods:** Quantitative RT-PCR was undertaken on RNA extracted from a number of murine arteries. Immunofluorescence was performed on single vascular smooth muscle cells using antibodies against the ERG1 expression product (Kv11.1). Single cell electrophysiology was performed on myocytes from portal vein and several different arteries, complimented by isometric tension recordings. Proliferation assays were undertaken on smooth muscle cells isolated from femoral arteries.

**Results:** ERG1 transcripts were detected in all murine blood vessels, and Kv11.1 immunofluorescence was observed in all smooth muscle cells. However, K^+^ currents with properties consistent with ERG channels were only recorded in portal vein myocytes. Moreover, ERG channel blockers (E4031 or dofetilide, 1 μM) failed to depolarize carotid arteries or produce contraction. Proliferation of arterial smooth muscle cells was associated with a marked increase in ERG1 expression and ERG blockers suppressed proliferation significantly.

**Conclusions:** These data reveal that arterial blood vessels express ERG channels that appear to be functional silent in contractile smooth muscle but contribute to proliferative response.

## Introduction

The late repolarizing phase of the ventricular action potential is dictated by K^+^ flux through voltage-dependent channels encoded by type 1 *ether-a-go-go* related genes (ERG1 or KCNH2) and mutations to this gene underlie type 2 long QT syndrome arrhythmias (Curran et al., 1996). Blockade of the hERG encoded channel (Kv11.1) underlie the majority of acquired arrhythmias. Two major isoforms of ERG1 have been identified in mammalian hearts (Lees-Miller et al., 1997; London et al., 1997; Pond et al., 2000), a full length variant (ERG1a) and a 340 amino acid N-terminal truncated ERG1 (ERG1b; Lees-Miller et al., 1997; London et al., 1997). Over-expression of both isoforms produces K^+^ currents with distinctive voltage-dependent kinetics due to a dominating C-type inactivation (Smith et al., 1996; Spector et al., 1996) and both isoforms are now considered to contribute to the native cardiac current (Larsen et al., 2008; Sale et al., 2008). Two other ERG genes (KCNH6 and 7, encoding for ERG 2 and 3 protein, respectively) exist, which are predominantly expressed in the central nervous system. In addition to the regulation of membrane potential, expression of ether-a-go-go genes and ERG have been implicated in cellular proliferation and oncogenesis (Babcock and Li, 2013).

In addition to the heart, hERG channels have been identified in several cell types, including visceral smooth muscle (for a review, see Vandenberg et al., 2012). ERG1 expression has been identified in murine portal vein and single cell electrophysiology revealed K^+^ currents with distinctive ERG kinetics that were inhibited by the ERG blockers dofetilide, E-4031, or rBekm-1 (Ohya et al., 2002; Yeung and Greenwood, 2007). However, nothing is known about the expression of ERG in arterial preparations and whether Kv11 channels contribute functionally to smooth muscle activity in these blood vessels. Consequently, we used quantitative PCR and immunofluorescence techniques, in combination with single cell electrophysiology and whole tissue isometric tension recordings, to explore the expression and the possible functional role of ERG1-3 in a number of arterial blood vessels.

## Materials and methods

### Experimental models

All experiments were performed in accordance with the Animals Act (1986) and St George's Animal Welfare Committee approval under Project license PPL 70/8512. Six to eight weeks old female BALB/c mice were killed by intraperitoneal injection with pentobarbitone, in accordance with schedule 1 of the United Kingdom Animals Act (1986) and conforms with the Guide and Care of Laboratory Animals published by the US National Institutes of Health (NIH Publication No. 85-23, 1996). For studies looking at the proliferative smooth muscle, arteries were taken from blood pressure normal (BPN) mice (Jackson Laboratories, Bar Harbor, ME USA), that have been used previously for such studies (e.g., Cidad et al., 2012). Mice were killed by decapitation after isofluorane anesthesia using protocols approved by the ethical committee of the University of Valladolid and in accordance with the European Community guiding principles. Blood vessels were excised and immediately placed into RNA Later (Ambion) for RNA extraction or Krebs for cell dispersal. Human Embryonic Kidney cells (HEK293) were used for immunofluorescence studies. Cells were transiently transfected with a plasmid encoding for mouse ERG using lipofectamine 2000 (ThermoFisher, Paisley, UK), according to manufacturer's instructions.

### Quantitative polymerase chain reaction

Total RNA from mouse arteries were extracted using the method described previously (Ohya et al., 2002; Yeung et al., 2007). One microgram of RNA were reversed transcribed using the reverse transcriptase (RTase) SuperScript® II RNase H- (Invitrogen, UK). All samples had a respective RT- control, i.e., no RTase was put into the sample. The following PCR primers were used.

mERG1 (GenBank accession no. AF012868): 2,145–2,272, amplicon = 128 bp (conserved region for both full length ERG1a and the N-terminal truncated ERG1b): 5′-CCCCTCCATCAAGGACAAGT-3′, 5′-TGAGCATGACACAGATGGAG-3′; mERG1a (AF012868): 369–533, amplicon = 165 bp: 5′-CCTCGACACCATCATCCGCA-3′, 5′-AGGAAATCGCAGGTGCAGGG-3′; mERG2 (NM_001037712), 328–468, 141 bp: 5′-GTGGATGTGGTCCCTGTGAA-3′; 5′-AGAGCCCAGGAAGCTGTGTG-3′; mERG3 (AJ291608), 3,012–3,180, 169 bp: 5′-GCCCGGGCTCAACCTGAAGA-3′, 5′-TGGCCTGGATGTCCGTTGTC-3′; glyceraldehyde 3-phosphate dehydrogenase (mGAPDH, M32599): 492–599, amplicon = 108 bp: 5′-CCTGCACCACCAACTGCTTA-3′, 5′-TCTTCTGGGTGGCAGTGATG-3. Real time quantitative PCR was performed with the use of Syber Green chemistry on an ABI 5700 sequence detector (PE Biosystems). To study the changes on genes expression on contractile vs. proliferative VSMCs the following TaqMan® Assays (Applied Biosystems) were employed: (1) ERG1—Kcnh2-Mm00465370_m1; (2) rRNA 18s—18S-Hs99999901_s1 (endogenous control). ERGs mRNA levels were normalized to the housekeeping genes GAPDH or 18S and expressed using the 2^−ΔCt^ or 2^−ΔΔCt^ formulae.

### Cell dispersal

Single vascular smooth muscle cells (VSMCs) were obtained from portal vein (PV) and various arteries by enzymatic digestion in physiological saline solution (PSS) containing (in mM) 60 NaCl, 6 KCl, 85 Na glutamate, 10 glucose, 10 HEPES, 2 MgCl_2_ 0.1 CaCl_2_, adjusted to pH 7.4 with NaOH. The tissues were bathed in 1 mg/ml papain (Worthington, USA) and 1 mg/ml DTT (Sigma, UK) made up in PSS containing 0 μM Ca^2+^ and maintained at 37°C for 8.5 min. They were then transferred to another enzyme mixture containing 0.5 mg/ml collagenase F (Sigma, UK) and 0.5 mg/ml collagenase H (Sigma, UK) made up in PSS containing 10 μM Ca^2+^ and also maintained at 37°C for 8.5 min. Tissues were then gently washed then triturated with ~150 μl ice cold PSS containing 50 μM Ca^2+^.

### Immunofluorescence

VSMCs and HEK293 cells were fixed with 3% paraformaldehyde solution at 22–24°C (RT) for 10 min, treated with 0.1 M glycine for 5 min and incubated in blocking solution (PBS containing 0.1% Triton X-100 and 1% bovine serum albumin) for 1 h at RT. Cells were then incubated overnight at 4°C with a goat polyclonal anti-HERG antibody (Santa Cruz, USA, dilution 1:100) or a rabbit polyclonal anti-HERG antibody (Abcam, Cambridge, UK, dilution 1:100), and mouse monoclonal anti-α-smooth muscle actin antibody (dilution 1:1,000, Sigma, Dorset, UK). Samples were then washed with PBS and incubated for 1 h with donkey anti-goat or anti-rabbit secondary antibody conjugated to Alexa Fluor 568 and donkey anti-mouse secondary antibody conjugated to Alexa Fluor 488 (dilution 1:100, ThermoFisher, Paisley, UK). All antibodies were diluted in blocking solution. Subsequently, samples were washed with PBS and analyzed using a Zeiss LSM 510 Meta argon/krypton laser scanning confocal microscope (Carl Zeiss, Jena, Germany).

### Electrophysiology

ERG channels exhibit distinctive voltage-dependent kinetics that are the product of a rapid inactivation dominating a slower activation and deactivation. Similar to previous studies on mouse portal vein smooth muscle cells (Ohya et al., 2002; Yeung and Greenwood, 2007) we recorded whole cell K^+^ currents using the ruptured patch configuration. Cells bathed in an external solution containing 5 mM KCl were held at −50 mV and stepped to +40 mV to activate voltage-dependent K^+^ channels and then repolarized to various potentials from −120 to −20 mV (see Yeung and Greenwood, 2007). In a further series of experiments cells were bathed in an external solution containing 140 mM KCl plus 10 mM tetraethyl ammonium and 5 mM 4-aminopyridine to block non-ERG K^+^ channels held at 0 mV and stepped to potentials between +20 and −120 mV (see Ohya et al., 2002). With this protocol ERG channels exist in an inactivated state at the holding potential and progressive hyperpolarization relieves the inactivation and allows the channel to close resulting in the distinctive hooked kinetics (e.g., Smith et al., 1996; Spector et al., 1996; Ohya et al., 2002). The external solution for voltage-clamp experiments contained (in mM): 126 NaCl, 5 KCl, 10 HEPES, 11 glucose, 1 MgCl_2_, and 0.1 CaCl_2_, adjusted to pH 7.2 with NaOH. Where the external solution contained 140 mM KCl no NaCl was present; all other constituents were identical, adjusted to pH 7.2 with KOH. The pipette (internal) solution contained (in mM) 130 KCl, 3 ATP Na^+^ salt, 0.1 GTP, 10 HEPES, and 1 MgCl_2_, adjusted to pH 7.2 with KOH. All drugs were made up to 100 mM stock solutions with either dimethyl sulfoxide (DMSO) or H_2_O.

### Isometric tension recordings

Carotid arteries and aorta (CA and TA, respectively) were cleaned of fat and 2 mm segments were mounted on 40 μm tungsten wires in a small vessel myograph (DMT, Aarhus), which contained 5 ml Krebs solution containing (in mM) 125 NaCl, 4.6 KCl, 2.5 CaCl_2_, 15.4 NaHCO_3_, 1 Na_2_HPO_4_, 0.6 MgSO_4_, and 10 glucose, maintained at 37°C and continuously aerated with 95% O_2_/5% CO_2_. Following equilibration, CA were normalized to a tension equivalent to that generated at 90% of the diameter of the vessel at 100 mmHg (Mulvany and Halpern, 1977). Segments of aorta were set to a resting tension of 10 mN based on previous optimization studies (Yeung et al., 2007) Tissues were then bathed in Krebs solution containing 60 mM KCl for 5 min, repeated twice. Changes in tension were acquired using PowerLab and Chart (version 5, ADInstruments, UK).

### Membrane potential recording in whole arteries

Membrane potential was measured continuously from whole CA mounted in a wire myograph using borosilicate microelectrodes (1.2 mm OD 0.69 I.D, World Precision Instruments, USA) that had resistances between 80 and 120 MΩ when filled with 2M KCl. Membrane potential (mV) was measured using an amplifier (NL-102, Digitimer, United Kingdom) and recorded on a using Powerlab 4/30 running Labchart 5 (AD instruments, Australia). Electrode entry into a vascular smooth muscle cell was determined by an abrupt drop in voltage, followed by a sharp return to baseline on exit, with a minimal change (no more than 10%) in resistance.

### Smooth muscle cell culture and proliferation

Dissected femoral arteries were cleaned of connective and endothelial tissues, and placed in a 35 mm culture dish covered with 2% gelatin (bovine skin type B, Sigma) in DMEM supplemented with 20% FBS, penicillin-streptomycin (100 U/ml each), 5 μg/ml fungizone, and 2 mM L-glutamine (Lonza) at 37°C in a 5% CO_2_ humidified atmosphere. When cells reached confluence, they were detached and seeded in a new culture plate at a 1/3 density in D-MEM medium supplemented with 5% FBS, penicillin-streptomycin (100 U/ml each), 5 μg/ml fungizone, L-glutamine (2 mM), Insulin (5 μg/ml), bFGF (2 ng/ml), and EGF (0.5 ng/ml). VSMCs were subjected to several passages without showing morphological changes. For proliferation assays, femoral VSMCs at passages 3–8 were seeded onto 12 mm diameter poly-l-lysine coated coverslips placed in 12 mm wells at a density of 25,000 cells/well and allow to attach in control medium (5% FBS). Afterwards, cells were synchronized in serum free (SF) medium during 48 h. Then, control media, alone or in combination with various ion channel blockers/activators, was added. Twenty-four hours post treatment, the percentage of cells at the S phase was quantified using EdU (5-ethynyl-2′-deoxyuridine) incorporation for an additional 6 h with a commercial kit (Click-iT® EdU Imaging Cell Proliferation Assay, Invitrogen). Finally, cells were incubated with Hoechst before mounting with Vectashield (Vector Laboratories Inc., Burlingame, CA). EdU incorporation was visualized with an immunofluorescence microscope (Nikon) at the corresponding wavelength depending on the Alexa Fluor® used and expressed as the percentage of the total cell number stained by Hoechst. In each experiment, this percentage was obtained from the average of 10–20 different fields per coverslip, and triplicates were made for each condition.

### Statistics

All data are expressed as mean ± s.e.m. One- or two-way ANOVA test followed by Dunnett's or Tukey's *post-hoc* analysis, and Student's *t*-test were used to determine statistical significance between groups, according to the different experiments, using Graph Pad Prism software. Differences were considered statistically significant when *p* < 0.05.

## Results

### Kv11.1 is expressed in vasculature

**Figure 1:**
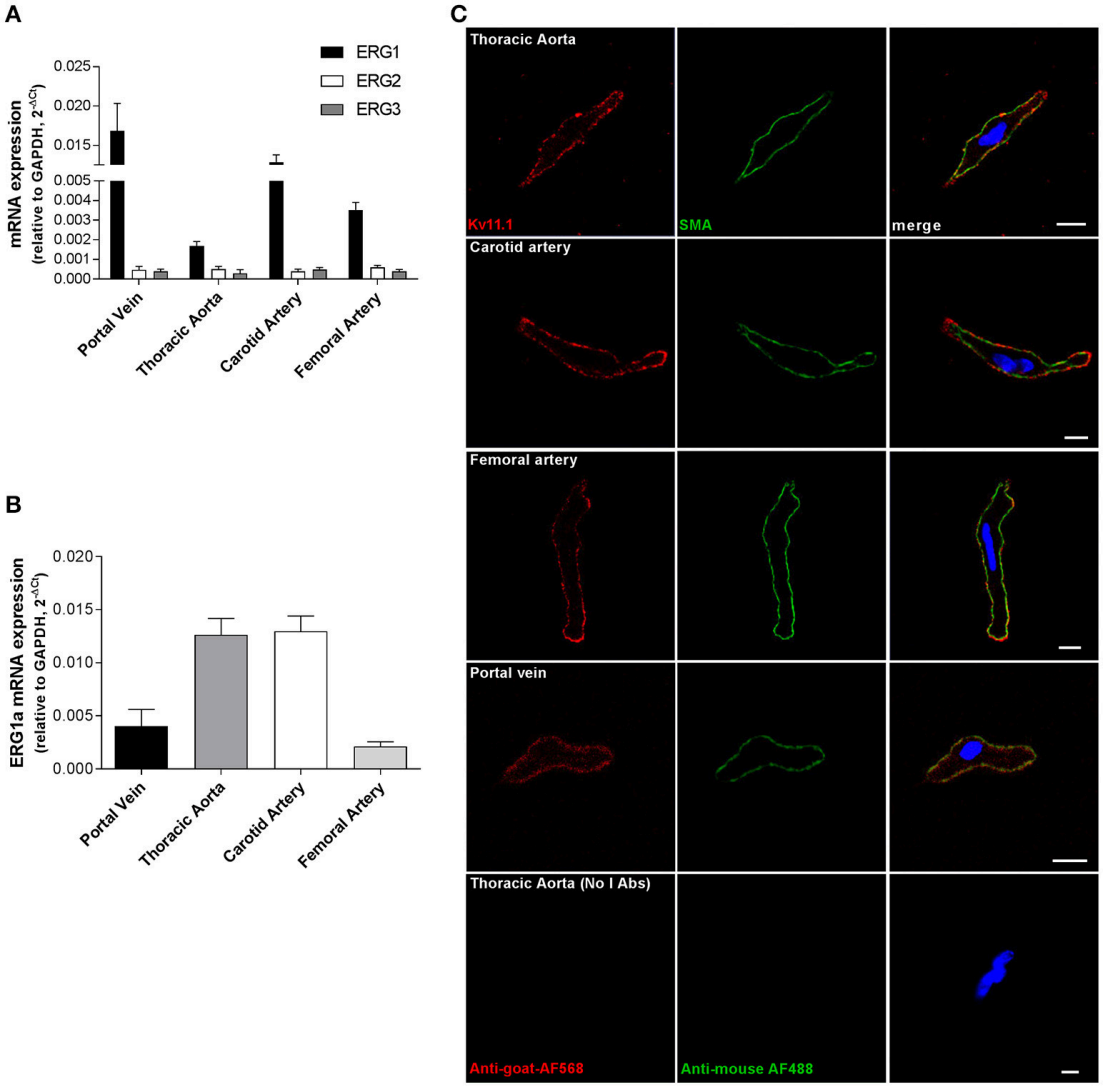
Expression of ERG in mouse vascular smooth muscle. **(A,B)** Results of quantitative PCR experiments showing expression of ERG1, 2 and 3 **(A)** and full length ERG1a isoform **(B)** normalized to GAPDH expression in a range of vascular beds. Each column is the mean of 4 tissues ± s.e.m. Data are expressed using the 2^−ΔCt^ formula **(C)** Fluorescent images of smooth muscle cells from different vessels labeled with Kv11.1 antibody (left column) and smooth muscle actin (SMA, middle column). Secondary antibodies only (no primary Abs) are shown at the bottom. Each image is representative of 3 separate dispersals. Scale bar = 5 μM.

To date evidence for ERG expression in blood vessels is confined to the mouse portal vein so quantitative PCR experiments were undertaken on a range of murine arterial vessels. Transcripts encoding for ERG1 were detected in aorta, carotid and femoral arteries, as well as in portal vein (Figure 1A). Aorta and carotid artery expressed higher levels of the full-length isoform (ERG1a) than portal vein and femoral artery (Figure 1B). In contrast, ERG2 and ERG3 mRNA levels in these vessels were negligible (*n* = 4 per vessel, Figure 1A). ERG2 and ERG3 transcripts were also undetectable in mesenteric and pulmonary arteries (data not shown). Immunofluorescence experiments were undertaken to look at the expression and the cellular localization of Kv11.1 in myocytes isolated from murine aorta, carotid and femoral arteries, and portal vein, using antibodies that specifically detected Kv11.1 in HEK293 expressing mERG, but not in untransfected cells (Supplemental Figure 1A). Smooth muscle cells were identified by staining for α-smooth muscle actin, which adopted a peripheral localization (Figure 1C, green pseudocolor) as reported previously (Shi and Sarna, 2013). Kv11.1 staining was detected in VSMCs from all arteries and from portal vein, which was predominantly located in or close to the cell membrane (Figure 1C, red pseudocolor). Similar results were obtained in immunofluorescence experiments with a different anti-Kv11.1 antibody (Supplemental Figure 1B).

### Functional role of Kv11 proteins in arterial myocytes

**Figure 2:**
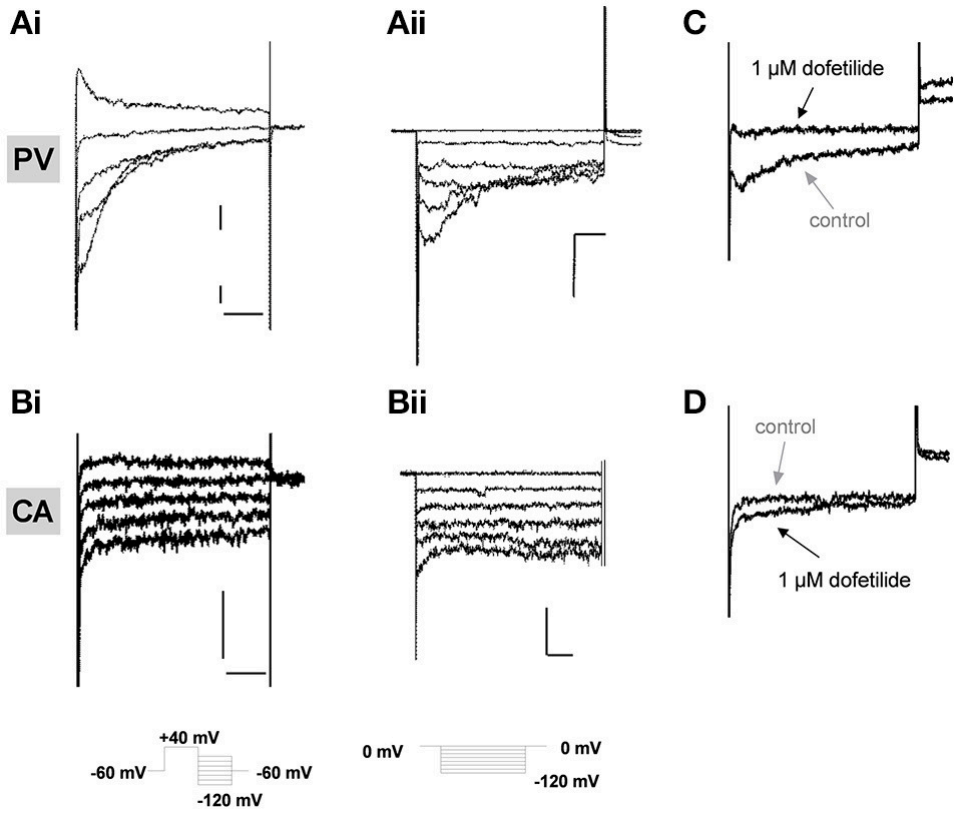
Electrophysiological recordings in isolated vascular myocytes. Examples of K^+^ currents recorded from portal vein (PV, **A**) or carotid artery (CA, **B**) myocytes in external solutions containing either 5 mM K^+^ **(i)** or 140 mM K+ **(ii)**. Voltage protocols shown as inserts. All scale bars represent 50 pA and 50 ms. Panels **(C,D)** show representative traces in control conditions (gray arrows) or in presence of 1 μM dofetilide (black arrows) on currents recorded at −100 mV in PV **(C)** and CA **(D)** following a step depolarization from −50 to +40 mV. All traces are representative of >5 cells from at least 3 animals.

In previous studies on murine portal vein myocytes Kv11 channel K^+^ currents were characterized using bathing solutions containing different concentrations of extracellular K^+^. In all conditions the current was identified by the distinctive, “hooked” voltage-dependent kinetics produced upon repolarization from a depolarizing test step, as the channels recover from inactivation and then close, and the sensitivity to known Kv11 channel blockers dofetilide and E4031 (Ohya et al., 2002; Yeung and Greenwood, 2007). We recorded similar currents with hooked kinetics in portal vein cells bathed in external solutions containing 5 mM K^+^ and 140 mM K^+^, which were abrogated by 1 μM dofetilide (Figures 2A–C). Figure 2B shows that in contrast to portal vein myocytes, “hooked” currents were not present in carotid artery myocytes from at least 5 different animals regardless of whether the cell was bathed in an external solution containing 5 mM (*n* = 15), 35 mM (*n* = 10), or 140 mM KCl (*n* = 6). A similar lack of distinctive, “hooked” currents was observed in aortic myocytes from 10 different animals (data not shown). The inhibitory effect of dofetilide seen in portal vein myocytes was not observed in carotid artery cells (Figure 2D, representative of 5 such records). These data suggest that Kv11 channel currents are not present in carotid or aortic smooth muscle cells.

### Effects of ERG channel activators

**Figure 3:**
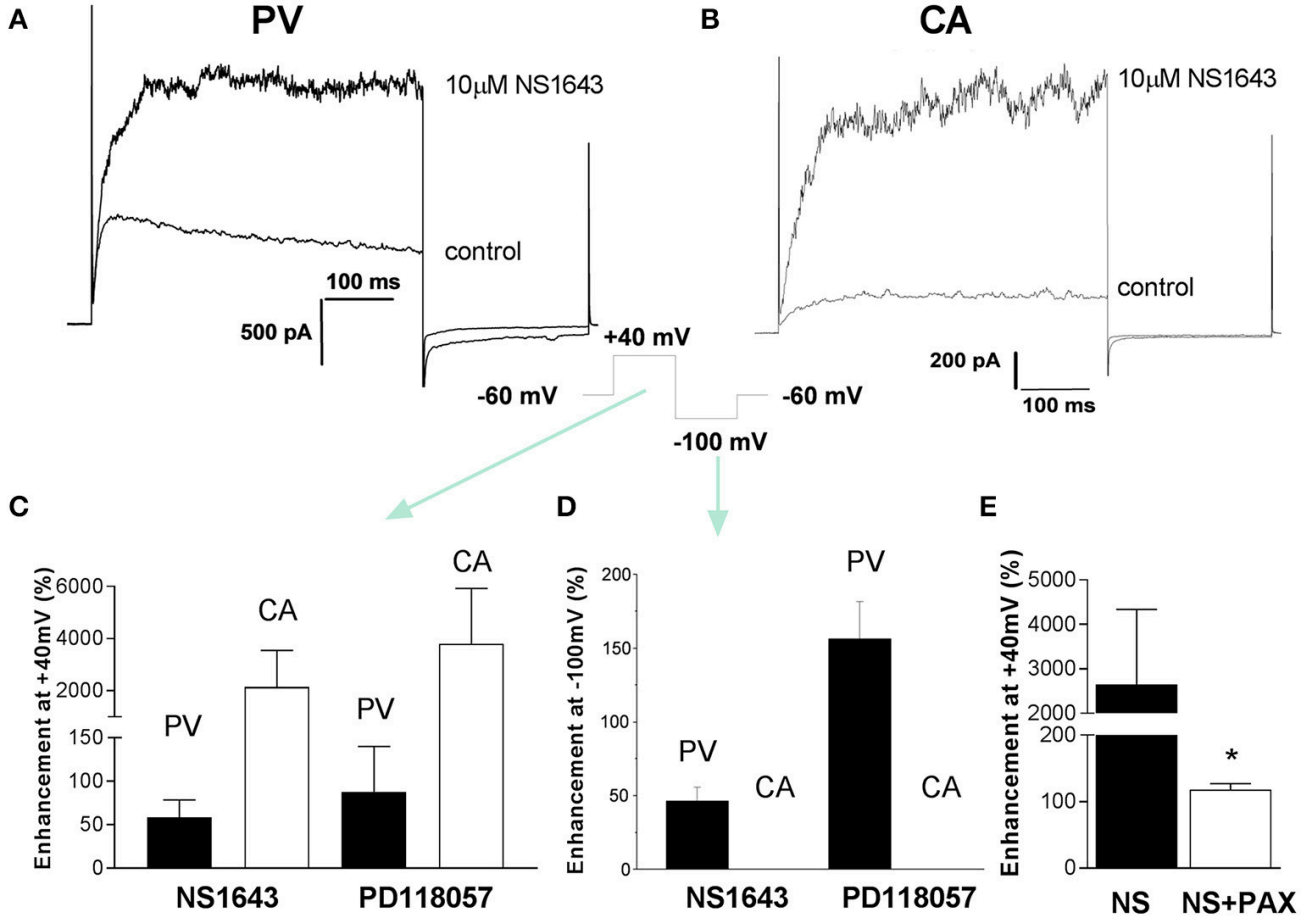
Effect of ERG channel activators on membrane currents. Examples of K^+^ currents evoked by membrane depolarization to +40 mV from −60 mV in the absence and presence of 10 μM NS1643 recorded from a PV myocyte **(A)** and CA myocyte **(B)**. Cells were repolarized to −100 mV after the test step to +40 mV. The mean increase in currents at +40 and −100 mV produced by two structurally different ERG channel activators (10 μM NS1643 and 3 μM PD118057) is shown in panels **(C,D)**, respectively. Each panel is the mean of 4–7 cells. Black columns = PV and white columns = CA. Panel **(E)** shows the effect of 1 μM paxilline on NS1643-stimulated currents at +40 mV in mCA myocytes (*n* = 8). Data are expressed as mean ± s.e.m. ^*^p < 0.05 according to Student's *t*-test.

Recently a number of different compounds that activate ERG-encoded K^+^ channels have been synthesized, including PD-118057 and NS1643 (Zhou et al., 2005; Casis et al., 2006; Hansen et al., 2006). It is possible that Kv11 channels are functional in arterial myocytes but operate at very low levels of activity. Consequently, experiments were undertaken to ascertain the impact of the ERG channel activators, NS1643 and PD-118057, on membrane currents in carotid artery myocytes. NS1643 (10 μM) augmented outward K^+^ currents at +40 mV in portal vein myocytes, which was associated with a marked increase in the amplitude and a slowing of the decay of the tail currents recorded upon repolarization to −10 mV (Figures 3A,C,D) from 52 ± 3 to 120 ± 28 ms (*n* = 4). A second ERG activator, PD118057 (3 μM), produced similar effects consistent with previous study in portal vein smooth muscle cells (Yeung and Greenwood, 2007). In carotid artery myocytes NS1643 had a negligible effect on the current recorded at −100 mV (change from 9 ± 1.8 to 15 ± 2 pA, *n* = 5) but increased the outward current considerably (Figures 3B–D). The stimulatory effect of NS1643 on current measured in carotid artery was not sensitive to 1 μM dofetilide (data not shown) but was inhibited by paxilline (1 μM), a blocker of large conductance, Ca^2+^-activated K^+^ channels (BK_Ca_; Figure 3E). PD118057 (3 μM) also had negligible effect on currents recorded at −100 mV but enhanced currents at +40 mV (Figures 3C,D) that was reversed completely upon application of paxilline (138.7 ± 27.2% inhibition *n* = 3, data not shown). These data suggest that in arterial myocytes, the ERG activators NS1643 and PD-118057 stimulated large conductance Ca^2+^-activated K^+^ channels but did not resurrect dormant ERG channels.

### Membrane potential and isometric tension recordings in whole arteries

**Figure 4:**
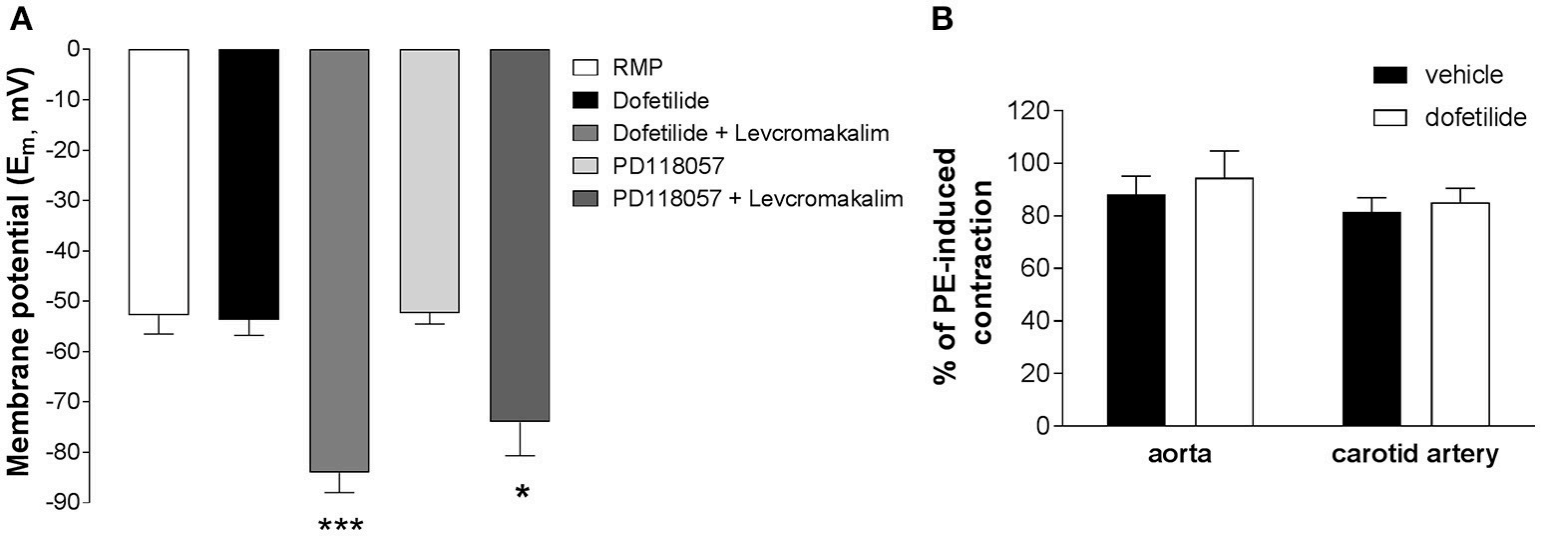
Effect of ERG channel modulators on membrane potential in whole carotid arteries. **(A)** Mean membrane potential (E_m_) in carotid arteries in the absence and presence of ERG blockers (1 μM dofetilide) and activators (3 μM PD118057). The ATP-sensitive K^+^ channel activator levcromakalim (10 μM) was applied as a control. Each column is the mean of 4–8 arteries ± s.e.m. ^*^p < 0.05, ^***^p < 0.001 according to one-way ANOVA. **(B)** Lack of effect of 1 μM dofetilide on contractions induced by 10 μM phenylephrine in mouse aorta or carotid artery (mean ± s.e.m, *n* = 6). Statistical analysis has been performed using Student's *t*-test (*p* > 0.05, non significant).

Whilst carotid artery and aortic myocytes lacked dofetilide-sensitive K^+^ currents with characteristic “hooked” kinetics, this did not entirely negate the possibility of a functional role for ERG channels in an intact tissue system. In sharp microelectrode studies on whole carotid arteries neither dofetilide (1 μM) nor the Kv11.1 activator PD-118057 (3 μM) affected the resting membrane potential (control: −52.8 ± 3.7 mV; dofetilide: −53.7 ± 1.2 mV; PD-118057: −52.3 ± 2.2 mV) whereas addition of the K_ATP_ activator levcromakalim (10 μM) produced marked hyperpolarization (dofetilide+levcromakalim: −84.0 ± 3.9 mV; PD-118057+levcromakalim: −74.0 ± 6.7 mV; Figure 4A). Kv11.1 channel blockers increase contractile activity in a number of visceral smooth muscle as well as the portal vein (Ohya et al., 2002; Farrelly et al., 2003; Parr et al., 2003; Mewe et al., 2008; Greenwood et al., 2009). In contrast, dofetilide (1 μM) did not affect resting tone in carotid arteries (*n* = 8) and did not augment the contractile response to 10 μM phenylephrine both in aorta and carotid artery (Figure 4B). These data suggest that Kv11.1 proteins have little functional effect in arterial smooth muscle.

### Kv11.1 activation promotes VSMCs proliferation

**Figure 5:**
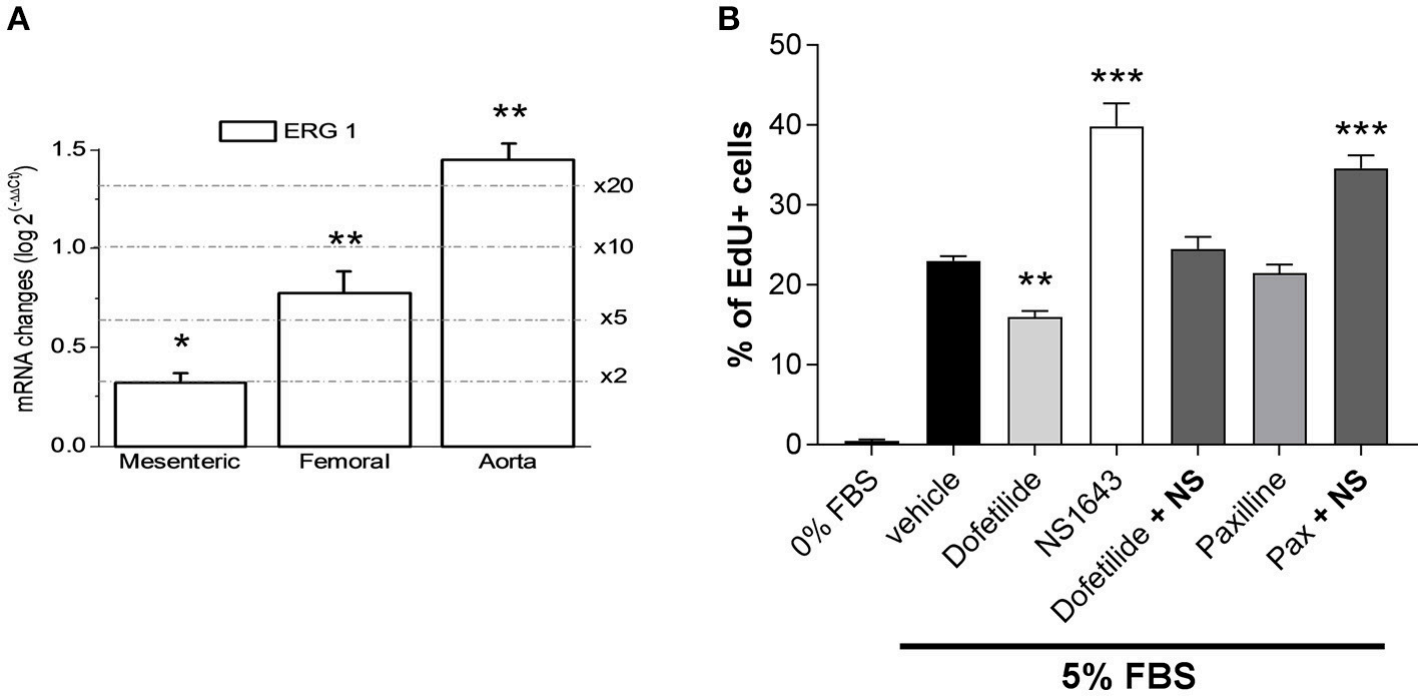
Changes in the expression pattern of ERG channels in proliferating arterial smooth muscle. **(A)** Differences in expression of ERG1 in cultured femoral VSMCs were calculated using 18S as housekeeping gene and the contractile VSMCs expression levels as calibrator, using the 2^−ΔΔCt^ formula, where ΔΔCt = ΔCt proliferative −ΔCt contractile and 2^−ΔΔCt^ represents the expression fold change. Each bar is the mean of 6–10 data from 3 to 5 assays in duplicate for each condition. ^*^p < 0.05; ^**^p < 0.01; ^***^p < 0.001, according to Student's *t*-test. **(B)** Proliferation rate (measured as % of cells incorporating EdU) of cultured femoral VSMC in the absence of FBS, or in culture media supplemented with 5% FBS in the presence of vehicle (control condition) or the indicated drug combinations. ^**^p < 0.01, ^***^p < 0.001 compared to control (vehicle), according to one-way ANOVA.

We next investigated the possible effects prompted by Kv11.1 modulation on arterial smooth muscle cells proliferation. We first explored the expression levels of ERG1 in proliferating smooth muscle cells from several vascular beds. To increase specificity, these experiments were carried out with validated Taqman probes. We compared ERG1 mRNA expression levels between fresh tissue from aorta, femoral and mesenteric samples (contractile VSMCs) and primary cultures of aortic, mesenteric and femoral VSMCs (proliferating cells). When using the abundance of the transcripts in the contractile VSMCs as the calibrator, we found significantly higher levels of ERG1 transcripts in cultured VSMCs in all preparations, ranging from a two-fold increase in mesenteric VSMCs to more than a 20-fold increase in aortic VSMCs in culture (Figure 5A). We explored if these changes in expression have some role in the phenotypic switch of VSMCs by determine the possible contribution of Kv11.1 to the proliferative response of the cultured VSMCs. Under the experimental condition used to induce proliferation, we found that smooth muscle cells isolated from femoral artery showed a better response to the proliferating stimuli when compared to other arteries (data not shown). Therefore, for the following experiments we focused on femoral VSMCs. Incubation with 1 μM dofetilide decreased serum-induced proliferation in cultured femoral by ~30% compared to vehicle-treated cells (from 22.46 ± 0.68 to 15.94 ± 0.97% of EdU positive cells). In contrast, the ERG channel activator NS1643 (10 μM) produced a ~153% increase in VSMCs proliferation (to 34.49 ± 2.1% of EdU positive cells), an effect that was fully abolished when applied in combination with dofetilide. Treatment with the BK_Ca_ blocker paxilline (1 μM) did not affect VSMCs proliferation (21.5 ± 1.0) and did not prevent NS1643-induced enhancement of VSMCs proliferation (34.6 ± 1.6) (Figure 5B).

## Discussion

ERG-encoded Kv11 channels were once considered to be the preserve of cardiomyocytes and neurons but recent studies have shown that these channels are effective regulators of membrane excitability and contractility in a number of smooth muscles including esophagus, jejunum, gall bladder, urinary bladder, epididymis, and uterus (Akbarali et al., 1999; Farrelly et al., 2003; Parr et al., 2003; Mewe et al., 2008; Greenwood et al., 2009). In each case the preparation in question is phasically active and generates action potentials. In these tissues application of Kv11 channel blockers such as dofetilide, E4031 or cisapride increases action potential duration, depolarizes the membrane potential and augments spontaneous contractions inherent to these vessels. These effects are associated with the abolition of a K^+^ current with distinctive “hooked” kinetics characteristic of ERG channel currents (Akbarali et al., 1999; Ohya et al., 2002). In addition, to these visceral smooth muscles ERG1 expression has also been identified in mouse portal vein where ERG channel currents have been characterized in low and high external K^+^ (Ohya et al., 2002; Yeung and Greenwood, 2007). Again, this tissue exhibits spontaneous contractility and action potential discharge. This study now reveals that a number of murine arteries, which do not exhibit spontaneous contractions and action potentials, express ERG1 but not ERG2 and ERG3, and that Kv11.1 subunits are expressed in VSMCs from several arterial beds, where they seem to localize at the plasma membrane levels. The expression and role of ERG channels in arteries that develop myogenic tone, such as cerebral artery, remain to be established. Our data also reveal a differential expression of the full-length isoform (ERG1a), with aorta and carotid artery showing higher levels compared to portal vein and femoral artery. As total levels of ERG1 are relatively higher in portal vein (Figure 1A), these data suggest that the expression of ERG1b isoform might be more abundant in this vessel, as described in previous studies (London et al., 1997; Pond et al., 2000). ERG1b lacks 373 amino acids of the 1a isoform and the N-terminus is replaced by a different 36-amino acid sequence (Vandenberg et al., 2012), and recent evidence suggest that incorporation of 1b isoform in the mature channels affect Kv11.1 trafficking and sensitivity to endogenous mediators and drugs (Phartiyal et al., 2008; Sale et al., 2008; Larsen et al., 2010).

### Lack of distinctive ion currents in arterial myocytes

In the present study no dofetilide-sensitive K^+^ currents with distinctive kinetics were recorded in carotid artery or aortic myocytes, in contrast to portal vein myocytes (present study and: Ohya et al., 2002; Yeung and Greenwood, 2007). This may have been due to the dissociation process as ERG1-encoded K^+^ channels are degraded by serine proteases (Rajamani et al., 2006). However, ERG channel currents have been recorded by various laboratories in smooth muscle cells isolated by similar dispersal techniques (such as portal vein, uterus, esophagus, Akbarali et al., 1999; Ohya et al., 2002; Greenwood et al., 2009) including the present study with portal vein myocytes. If this is the case then it would suggest that for some reason the arterial Kv11.1 protein is more susceptible to proteolytic degradation that the venous Kv11.1 protein.

### Functional effects

ERG channel blockers failed to depolarize carotid arteries and did not contract segments of aorta, carotid artery, or femoral artery unlike the effect of these agents in spontaneously active tissues described above. Dofetilide also had no effect on tone in rat aorta, mesenteric, and intralobar arteries (Doggrell and Nand, 2002) and does not raise mean arterial blood pressure in dogs or monkeys (Haushalter et al., 2008). Thus, we believe that ERG channels likely exist in arteries in a functionally silent state due to underlying processing or regulation. Interestingly, ERG channel blockers are spasmogenic in myometrium from non-pregnant mice but are ineffective in myometrium from late pregnant animals (Greenwood et al., 2009). This is associated with an inability to record ERG channel currents in myocytes dispersed from late pregnant myometrium, which are present in myocytes from non-pregnant tissues (Greenwood et al., 2009). This was not due to a decreased expression of Kv11.1 protein but was associated with an increase in KCNE2 and KCNE4, which encode for auxiliary subunits that alter the membrane abundance and open probability of ERG channels (Abbott et al., 1999; Mazhari et al., 2001; McCrossan and Abbott, 2004). Thus, the lack of ERG channel function in arteries may be due to a suppressive effect of the KCNE subunits expressed in the arteries, which we have shown to be highly variable throughout the vascular tree (Yeung et al., 2007). Alternatively, the different content of ERG1a and 1b subunits of the tetrameric Kv11.1 channel may have an impact on the membrane presentation of the mature channel, as co-assembly increases the amount of channels in the membrane and markedly increases current flow (Guasti et al., 2008). Portal vein myocytes, which exhibited dofetilide-sensitive currents with distinctive “hooked” kinetics expressed ERG1b at considerable levels (Ohya et al., 2002).

### Kv11 and smooth muscle proliferation

In contrast to the lack of functional effects in contractile arteries, ERG channel modulators had a marked impact on proliferating arterial smooth muscle cells, with dofetilide retarding serum-driven increase in cell number. Moreover, ERG1 expression increased in proliferating arterial smooth muscle cells. Although, we focused on femoral arteries as they responded better than other arteries in our experimental conditions, we observed a similar increase in ERG1 expression in other arteries as they switched from contractile to proliferative states (Figure 5A). The fact that the effects of NS1643 and dofetilide are mutually canceled when used in combination, together with the lack of effect of paxiline, a selective BK channel blocker, in preventing NS1643-induced increase of proliferation excludes off-target effects of the drugs, and in particular the contribution to these effects on proliferation of a modulation of BK channels by NS1643 as described above. These findings are in agreement with many observations that ERG1 (KCNH2) and the closely related EAG (KCNH1) have considerable oncogenic potential. Indeed, ERG1 and EAG transcripts are up-regulated in tumor cells, while pharmacological blockade or knock-down of ERG1 and EAG retards tumor cell proliferation (Arcangeli et al., 1995; Pardo and Stühmer, 2014). Our findings suggest that altered ERG1 expression may contribute to disease-related smooth muscle proliferation and implicates these ion channels as potential targets as inhibitors of tumor driven neovascularization.

### Limitations of the study

In the present study we have used vessels from different strains of mice. However, we checked that no differences in terms of ERG expression and function were observed in BALB-C mice and BPN mice prior to perform the proliferation experiments. Whilst we observed an increase in ERG1 transcript levels in proliferating VSMCs, we were unable to confirm these results by evaluating Kv11.1 protein expression. Moreover, the resting membrane potential was minimally affected by an ERG activator and blocker in these cells (~2 mV change). We were not able to record any ERG-mediated current in proliferating VSMCs. It should be noticed that similar results (i.e., lack of measurable currents) have been observed in other experimental paradigms evaluating the role of voltage-gated Kv7 channels in myoblasts differentiation, despite a clear functional effects exerted by Kv7 modulators (Iannotti et al., 2010). Consequently, whilst the effects of ERG modifying agents on cell number were clear, the specific involvement of ERG1 in VSMCs proliferation needs to be confirmed using more selective tools such as gene silencing. Moreover, additional experiments should be performed to rule out the role played by different ERG1 isoforms in this process.

## Conclusions

This study reveals that Kv11.1 (commonly known as ERG) is expressed in murine arteries, where localizes at the cell membrane of smooth muscle cells but without exerting any functional effect on vascular contractility and membrane excitability. However, Kv11.1 channel modulators had considerable effects on proliferating smooth muscle cells. The mechanisms that make these channels functionally silent in contractile smooth muscle and drive the increased ERG1 expression in proliferating arterial smooth muscle have not been yet elucidated and need to be investigated in future studies.

## Author contributions

VB, IG, MP designed of research, performed experiments, analyzed data, interpreted results of experiments, drafted manuscript, and prepared figures; PC, SY, AM, JL, SO performed experiments, analyzed data, and interpreted results. All Authors critically reviewed and approved the final version of the manuscript and agree to be accountable for all aspects of the work.

### Conflict of interest statement

The authors declare that the research was conducted in the absence of any commercial or financial relationships that could be construed as a potential conflict of interest.
