# Supplementary material for: Proliferative Role of Kv11 Channels in Murine Arteries
Source: Front Physiol. 2017 Jul 12;8:500. doi: 10.3389/fphys.2017.00500 (PMC5506201; doi:10.3389/fphys.2017.00500)
Supplement: Supplementary file 1 [file Presentation1.PDF]

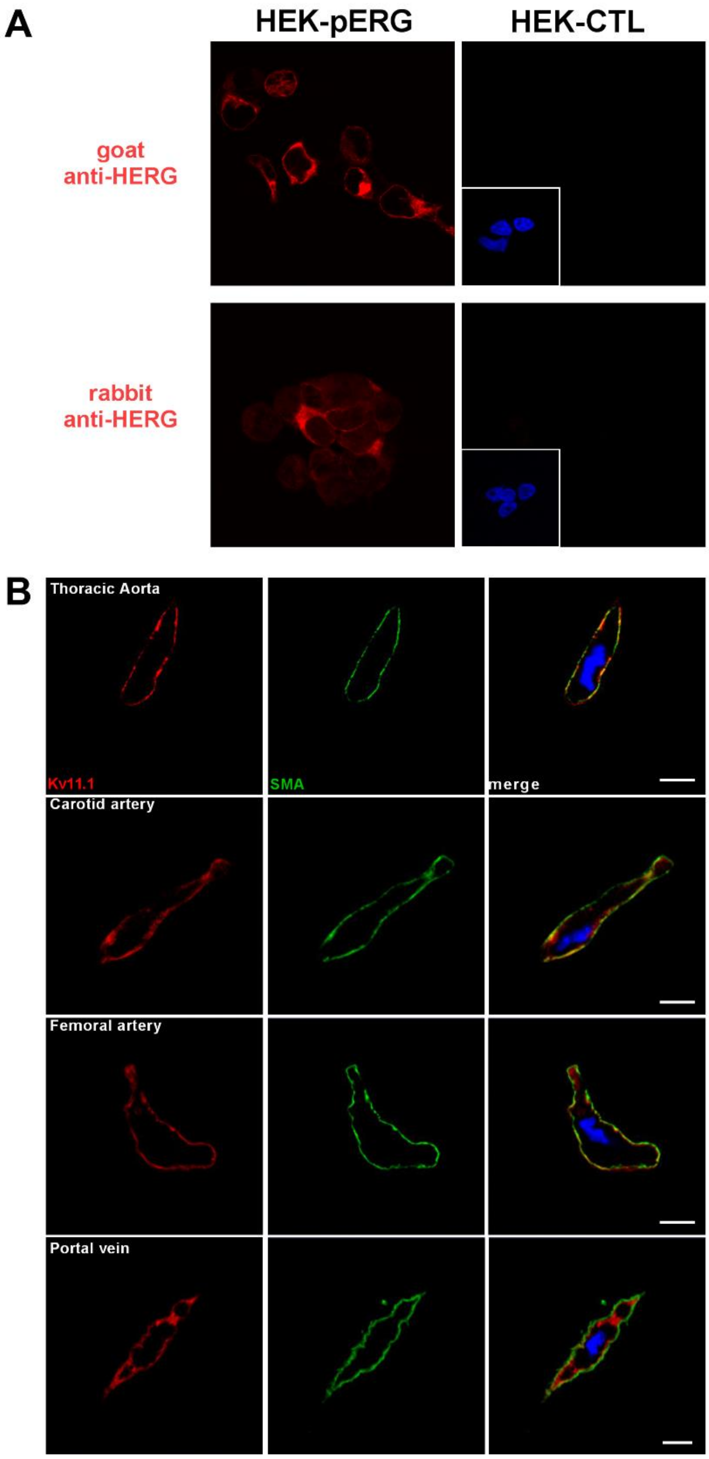

Supplemental Figure 1. Expression of ERG in transfected HEK cells and in mouse arterial smooth muscle. (A) Representative images of HEK cells transfected with a plasmid encoding for mERG (left panels) and stained for Santa Cruz goat anti-HERG antibody (top panel), or Abcam rabbit anti-HERG antibody (bottom panel). Non transfected cells (negative control) stained with the same antibodies are shown in the right panels. Insets shows nuclei in the experimental field where the image has been acquired **(**B) Fluorescent images of VSMCs from different vessels labelled with rabbit anti-HERG antibody (Kv11.1, Abcam, left column) and smooth muscle actin (SMA, middle column). Each image is representative of 3 separate dispersals. Scale bar=5µM.
